# Changing the name of the NBPF/DUF1220 domain to the Olduvai domain

**DOI:** 10.12688/f1000research.13586.2

**Published:** 2018-07-17

**Authors:** James M. Sikela, Frans van Roy

**Affiliations:** 1 Department of Biochemistry and Molecular Genetics, Human Medical Genetics and Neuroscience Programs, University of Colorado School of Medicine, Aurora, CO, 80045, USA; 2 Department of Biomedical Molecular Biology, Ghent University, Ghent, 9052, Belgium; 3 VIB-UGent Center for Inflammation Research, Ghent, 9052, Belgium

**Keywords:** DUF1220, NBPF, protein domain, human brain evolution, copy number, gene duplication, genome evolution, Olduvai Gorge

## Abstract

We are jointly proposing a new name for a protein domain of approximately 65 amino acids that has been previously termed NBPF or DUF1220. Our two labs independently reported the initial studies of this domain, which is encoded almost entirely within a single gene family. The name Neuroblastoma Breakpoint Family ( *NBPF*) was applied to this gene family when the first identified member of the family was found to be interrupted in an individual with neuroblastoma.

Prior to this discovery, the Pfam database had termed the domain DUF1220, denoting it as one of many protein domains of unknown function. It has been Pfam’s intention to use “DUF” nomenclature to serve only as a temporary placeholder until more appropriate names are proposed based on research findings.

We believe that additional studies of this domain, primarily from our laboratories over the past 10 years, have resulted in furthering our understanding of these sequences to the point where proposing a new name for this domain is warranted. Because of considerable data linking the domain to human-specific evolution, brain expansion and cognition, we believe a name reflecting these findings would be appropriate. With this in mind, we have chosen to name the domain (and the repeat that encodes it) Olduvai. The gene family will remain as *NBPF* for now. The primary domain subtypes will retain their previously assigned names (e.g. CON1-3; HLS1-3), and the three-domain block that expanded dramatically in the human lineage will be termed the Olduvai triplet.

The new name refers to Olduvai Gorge, which is a site in East Africa that has been the source of major anthropological discoveries in the early-mid 1900’s. We also chose the name as a tribute to the scientists who made important contributions to the early studies of human origins and our African genesis.

Protein domains are portable units within proteins that can serve important biological functions. They have been implicated in a broad range of key biological phenomena, including development, disease and evolution ^1, 2^. Here we jointly propose a new name for a protein domain of approximately 65 amino acids that has been previously termed NBPF ^3^ or DUF1220 ^4^. Our two labs independently reported the initial studies of this domain, which is encoded almost entirely within a single gene family. The name Neuroblastoma Breakpoint Family ( *NBPF*) was applied to this gene family when the first identified member of the family was found to be interrupted in an individual with neuroblastoma ^3, 5^. Prior to this discovery, the Pfam database had termed the domain DUF1220, denoting it as one of many protein domains of unknown function ^6^. It has been Pfam’s intention to use “DUF” nomenclature to serve only as a temporary placeholder until more appropriate names are proposed based on research findings. We believe that additional studies of this domain, primarily from our laboratories over the past 10 years, have resulted in furthering our understanding of these sequences to the point where proposing a new name for this domain is warranted.

Key findings relevant to assigning a name to the domain are as follows:

1. **The domain has been repeatedly linked with human-specific evolution.** The haploid human genome is estimated to encode approximately 300 copies of the NBPF/DUF1220 domain, while the copy number for other species is substantially lower: great apes 90–120, monkeys 30–40, and all other mammalian species 1–9 ^7, 8^. The increase in humans (at least 165 additional human copies) represents the largest human lineage-specific copy number increase of any coding region in the genome. These findings, involving the copy number of a protein coding domain, provide strong support for an involvement in human-specific evolution.
2. **The domain has been linked with human brain evolution and cognitive function.** Over the past 10 years we have published several papers on NBPF/DUF1220 protein domains and the *NBPF* gene family. These have implicated the copy number of the domain in human brain evolution ^7, 9– 11^, brain size-related phenotypes ^7, 9– 11^, brain disorders (autism/schizophrenia/micro- and macrocephaly) ^9, 12– 14^, and measures of cognitive function ^13, 15^. Also, our finding of a robust linear association between NBPF/DUF1220 copy number and brain size across primate species was confirmed by an independent study ^16^.

Given the above research findings, a new name for this protein domain that is related to human-specific evolution would be appropriate. We believe a name that would do this is “Olduvai” (ohl’-du-vi) (when necessary it can be abbreviated as “ODV”). This name refers to Olduvai Gorge, which is located in the rift valley of Eastern Africa. Olduvai has been the site of key paleoanthropological discoveries related to human origins and has been called “the Cradle of Mankind”, and “the Grand Canyon of Human Evolution” ^17^. Deposits at the gorge are estimated to cover a time span from 2.1 million to 15,000 years ago, and the fossil remains that have been identified there are thought to represent more than 60 hominins (members of the human lineage). These findings are believed to constitute the most continuous known record of human evolution over the past 2 million years, and the longest known archaeological record of the development of stone-tool industries. Olduvai Gorge was designated part of a UNESCO World Heritage site in 1979 ^18^. Just as the protein domain appears to be important to human-specific evolution, so too, Olduvai Gorge has provided key insights into human’s evolutionary origin. We believe both are central to the story of what made us human.

Finally, we have also chosen this name because it reflects an appreciation for the important contributions of the scientists who made major anthropological discoveries in Africa in the early/mid-20 ^th^ century that stimulated further research into human origins and our African genesis. While we are aware that naming a protein domain after a geographic location is unusual, we believe the name Olduvai is more than a place. Rather it has come to be thought of as a symbol of the efforts of our species to understand itself.

While we believe that the domain and repeat should now be called “Olduvai”, we also propose that, for now, the gene family name should remain *NBPF*. It is also worth noting that there is an Olduvai domain that is separate from those encoded by *NBPF* genes: a single, likely ancestral, copy of the domain is found within the *PDE4DIP*/myomegalin gene. In summary, the NBPF domain and DUF1220 domain will be termed the Olduvai domain, and the NBPF repeat and DUF1220 repeat will be termed the Olduvai repeat. The primary gene family that encodes these sequences will continue to be called *NBPF*, and the primary domain subtypes will retain established nomenclature (CON1-3, HLS1-3) ^8^. The three-domain block, composed of HLS1, HLS2 and HLS3 subtypes, that is tandemly repeated within several human *NBPF* genes and responsible for the great majority of additional human copies of the domain, will be called the Olduvai triplet.

The Olduvai domain hyper-amplification in the human lineage was one of the most extreme and rapid copy number expansions in the human genome, and we look forward to additional studies that may provide further insights into the role this protein domain family plays in human disease and evolution.
