# Supplementary material for: Changing the name of the NBPF/DUF1220 domain to the Olduvai domain
Source: F1000Res. 2018 Jul 17;6:2185. Originally published 2017 Dec 28. [Version 2] doi: 10.12688/f1000research.13586.2 (PMC5773923; doi:10.12688/f1000research.13586.2)
Supplement: Peer review history

# Peer review: Referee response for version 2

**DOI:** 10.5256/f1000research.16945.r36115

**Published:** 2018-07-25

**Authors:** Christian J.A. Sigrist

**Affiliations:** 1 Swiss Institute of Bioinformatics (SIB), Geneva, Switzerland

The authors have taken into account the reviewers remarks. I hope that this paper will encourage the NBPF and myomegalin communities to join efforts to unravel the function of the Olduvai domain and its role in the emergence of mankind.

I have read this submission. I believe that I have an appropriate level of expertise to confirm that it is of an acceptable scientific standard.

# Peer review: Referee response for version 2

**DOI:** 10.5256/f1000research.16945.r36117

**Published:** 2018-07-17

**Authors:** Alex Bateman

**Affiliations:** 1 European Molecular Biology Laboratory, European Bioinformatics Institute (EMBL-EBI), Cambridge, UK

I approve the new version of the manuscript.

I have read this submission. I believe that I have an appropriate level of expertise to confirm that it is of an acceptable scientific standard.

# Peer review: Referee response for version 1

**DOI:** 10.5256/f1000research.14757.r29429

**Published:** 2018-01-18

**Authors:** Elspeth A. Bruford

**Affiliations:** 1 HUGO Gene Nomenclature Committee (HGNC), European Bioinformatics Institute (EMBL-EBI), Hinxton, UK

This brief opinion article describes the authors' proposal to rename a domain found in a discrete group of proteins, which was originally named DUF1220 (domain of unknown function 1220) and subsequently reassigned as NBPF. The NBPF nomenclature referenced the fact that this domain is found in proteins encoded by a family of genes initially reported as the "Neuroblastoma BreakPoint Family"; however, as human gene nomenclature tries to move away from directly referencing specific phenotypes and conditions, the reference to neuroblastoma has been removed from the names for the NBPF genes, and they are now simply the "NBPF family".

 Interestingly, as well as this family the only other protein this domain is found in is the large "myomegalin" protein in mammals, encoded by the PDE4DIP gene. I think the fact that this is not mentioned anywhere in the article is perhaps an oversight on the part of the authors which could be remedied.

 It should be noted that the authors have already contacted PFAM to request this change and HGNC were also consulted and agreed this update would not create problems from a gene naming perspective. As we said then, we think it would be more informative for the new domain name to reflect functional information as opposed to something more esoteric, but the authors are not advocating any parallel change in the NBPF gene family nomenclature which is quite widely used for this family. A parallel change to the gene names would perhaps be more difficult to justify. Therefore we told the authors that we see no issue with their proposal.

 Importantly, this article represents the collaborative effort of two independent groups, who easily could be viewed as "rivals". Therefore this article effectively shows that such collaborations are possible and beneficial to the scientific community at large; this kind of activity should be encouraged wherever possible and I applaud the authors for making the effort to work productively together.

I have read this submission. I believe that I have an appropriate level of expertise to confirm that it is of an acceptable scientific standard.

# Peer review: Referee response for version 1

**DOI:** 10.5256/f1000research.14757.r29374

**Published:** 2018-01-17

**Authors:** Christian J.A. Sigrist

**Affiliations:** 1 Swiss Institute of Bioinformatics (SIB), Geneva, Switzerland

This paper proposes to change the name of a protein domain from NBPF (Neuroblastoma Breakpoint Family) to Olduvai, arguing that this new name illustrates the fact that the domain copy number has been linked with human evolution. Although, it is in principle not recommended to multiply the names for a domain, the fact that the change is submitted by two major players in the field makes the proposal acceptable as it should rapidly spread in future works on this promising domain. In addition, the limited distribution of the domain should facilitate its diffusion. However, it should be noted that although having a very limited distribution found in roughly two protein families, i.e. NBPF and myomegalin *(* *PDE4DIP* *)*, neither of the current names, NBPF or DUF1220, is commonly used by the myomegalin community. In this respect, I think that a paragraph presenting the myomegalin family and discussing the presence of a single Olduvai “repeat” amongst its members should be added prior to indexing.

 The previous name, NBPF, was derived from a breakpoint in the first member identified and was also applied to the protein family. In the future, the use of the new name Olduvai only for the repeat will avoid the confusion between NBPF designating both the protein family and the repeat.

I have read this submission. I believe that I have an appropriate level of expertise to confirm that it is of an acceptable scientific standard, however I have significant reservations, as outlined above.

# Peer review: Referee response for version 1

**DOI:** 10.5256/f1000research.14757.r29376

**Published:** 2018-01-02

**Authors:** Alex Bateman

**Affiliations:** 1 European Molecular Biology Laboratory, European Bioinformatics Institute (EMBL-EBI), Cambridge, UK

This manuscript describes a straightforward proposal to rename the NBPF/DUF1220 domain to the Olduvai domain. This is welcome because the domain has been referred to by two different names in the past.  The authors are the major players in the field, who have used the divergent nomenclature and thus will be able to ensure that the proposed naming does become the standard.  The authors had already contacted me to ask about having the domain renamed within the Pfam database, which has now been done (although not visible on the public website yet).  Perhaps calling this paper a proposal is a bit weak. The authors should consider renaming the article to “Changing the name of the NBPF/DUF1220 domain to the Olduvai domain”.

 There are no agreed upon standard naming schemes for protein domains or families. To date the most popular schemes are based on naming after one or more proteins that contain the domain, sometimes based upon the initial letters of these members.  A few domains are named after scientists.  I don’t recall any domains or families named after places.  However, the renaming makes sense within the context of the evolution of this domain and human evolution.

 I think it would be worth mentioning the reasons that the NBPF nomenclature is retained for the gene family. I understand this is to fit with the HGNC guidelines.

**Minor points**

 Please change PFAM to Pfam throughout the text.

I have read this submission. I believe that I have an appropriate level of expertise to confirm that it is of an acceptable scientific standard.
